# Visualization and Quantification of the Oral Hygiene Effects of Brushing, Dentifrice Use, and Brush Wear Using a Tooth Brushing Simulator

**DOI:** 10.3389/fpubh.2019.00091

**Published:** 2019-05-08

**Authors:** Ruth G. Ledder, Joe Latimer, Sarah Forbes, Jodie L. Penney, Prem K. Sreenivasan, Andrew J. McBain

**Affiliations:** ^1^Division of Pharmacy and Optometry, School of Health Sciences, University of Manchester, Manchester, United Kingdom; ^2^Colgate-Palmolive Company, Piscataway, NJ, United States; ^3^Department of Oral Biology, Rutgers School of Dental Medicine, Rutgers University, Newark, NJ, United States

**Keywords:** dental plaque, brushing simulator, typodont, biofilm model simulated plaque, brush wear

## Abstract

Approaches that reproduce dental hygiene regimens under controlled conditions have applications in preclinical research. We have applied standardized, reproducible brushing regimes to typodonts coated in simulated or biological plaques to assess the effects on tooth cleaning of toothbrush/dentifrice regimens. Replicated typodonts were coated with Occlude^TM^ or Glogerm^TM^ indicators to simulate plaque, and brushed reproducibly using a mechanical brushing simulator to compare the cleaning of occlusal surfaces before and after brushing with water or a dentifrice. An *in vitro* model using salivary inocula to cultivate oral biofilms on typodont surfaces was then developed to evaluate removal of disclosed plaque by new toothbrushes in comparison to toothbrushes with wear equivalent to 3 months of use. Analyses of typodonts brushed under controlled conditions significantly (*p* < 0.01) distinguished between brushed and unbrushed surfaces and between the use of water vs. dentifrice for the removal of simulated interproximal plaque (*p* < 0.05). New toothbrushes removed significantly (*p* < 0.05) more biological plaque from typodont surfaces than brushes that had been worn by repeated brushing. Through controlled and defined brushing of typodonts with simulated and biological plaques, the effectiveness of dental hygiene regimens was compared under reproducible conditions. Data indicate that the cleaning effectiveness of brushing was augmented by the addition of dentifrice and that new brushes were significantly more effective than brushes with substantial wear from previous use. Whilst we have focussed on the occlusal surfaces of molars and worn brushes, the method could be applied to a range of other tooth surfaces and oral hygiene regimens.

## Introduction

Accumulation of dental plaque is etiologically linked to the development of both dental caries (1–3) and periodontal disease (4–6) The mechanical removal of dental plaque plays an important role in the maintenance of oral health (7, 8) through the prevention of caries and periodontal disease (9, 10). Regular brushing with a toothbrush and a fluoridated dentifrice that may also include antimicrobials and other agents (11, 12) is a recommended and globally adopted method of controlling plaque (13). Brushing techniques differ between individuals for a variety of factors including duration, pressure and coverage (14). Such inter-individual variation may confound attempts to reproducibly identify and accurately quantify the effects of for example, brush type or dentifrice on plaque removal. Clinical studies indicate that most adults only reduce their plaque scores by up to 58% following brushing (15) after refraining from oral hygiene for 48 h. Therefore, many adults are living with significant amounts of dental plaque despite brushing twice a day, which is evidenced by continued high incidence of dental disease (16). The global incidence of dental disease further suggests poor compliance with recommended effective regimens (17, 18).

Since new toothbrush designs may be developed in order achieve improved cleaning efficacy, there is a need for methods to facilitate the accurate assessment of their effectiveness. Preclinical testing is a useful option either as a substitute for clinical studies, or to support improved design and targeting of clinical trials. Pre-clinical studies of brushing effectiveness date back to the 1970s where for example, Arnold and Trost (19) used acrylic tooth models and a dye to investigate brushing effectiveness using a mechanical brushing simulator. In 1979, an interproximal model was developed using registration tape wrapped around artificial dentition. After each brushing sequence (which was varied according to pressure, technique, and tooth shape), a print was left on the tape, which allowed inter-proximal reach to be ascertained (20). In the 1990s, a typodont model stained with blue ethyl cellulose was employed by Rawls et al. (21) to assess plaque removal using of a color-removal index. In other reports (22, 23) two different mechanized approaches were used to assess the brushing of typodonts dyed with chromogenic stain, which achieved greater standardization and efficacy of comparative analyses. Brushing simulators capable of running programmable three-dimensional brushing patterns are now available. These systems have been previously used with typodonts in conjunction with a water-based dye (23, 24), together with digital image analysis (25) to compare the efficacy of plaque removal between battery-operated toothbrushes. Three-dimensional lasers have also been employed to assess artificial plaque removal from a typodont following wear of toothbrush heada (26). Programmable mechanical brushing simulators are now a well-established method for the analyses of toothbrush efficacy (27, 28).

Whilst it has been reported that brushing without a dentifrice removes significant amounts of plaque (29–31), the use of a dentifrice in Western societies is considered to be a fundamental part of oral hygiene regimes (32) and is recommended by the American Dental Association (ADA). The ADA claims that the use of dentifrice enhances the plaque removal of the toothbrush. However, in conflicting reports, van der Weijden and Slot (32) and Valkenburg et al. (33) have suggested that using dentifrice does not necessarily lead to a greater removal of dental plaque.

With respect to the hygienic benefits of a toothbrush with a low wear index, some previous studies (34, 35) have concluded that worn toothbrushes are less efficient at plaque removal and the control of gingivitis than toothbrush without significant wear. Additionally, worn toothbrushes are reportedly more likely to harbor potential oral pathogens including *Streptococcus mutans* (36).

The aim of the current study was to develop a method by which to visualize and quantify simulated and biological plaque removal *in vitro* in a reproducible manner, using simulated or real plaque. The method was then applied to differentiate the effects of water vs. dentifrice in a silica base and worn vs. unworn brushes.

## Materials and Methods

### Development of a Reproducible Brushing Model

**Figure 1:**
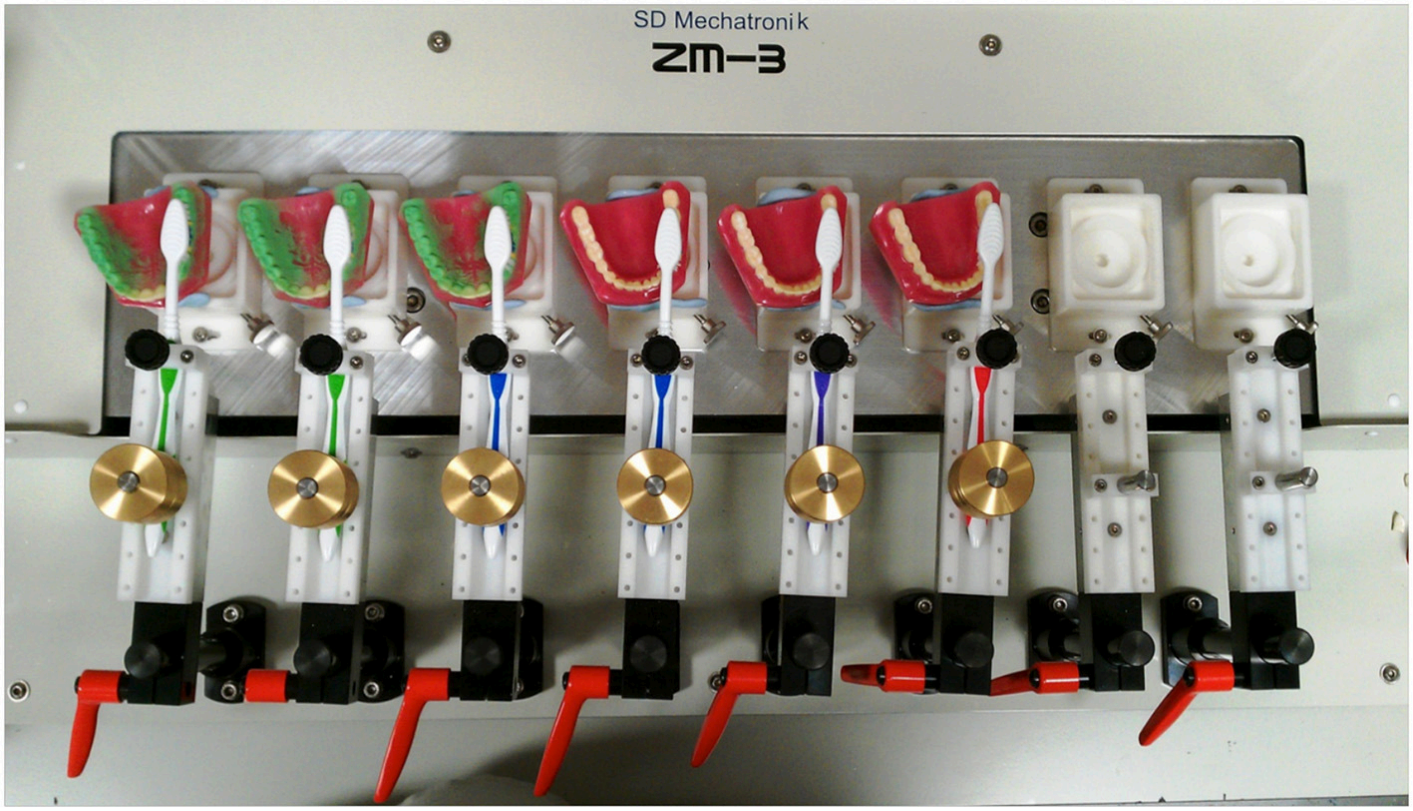
A typical experimental set-up. Up to eight typodonts were coated with simulated plaque and mounted with brushes on the brushing simulator. Typodonts were brushed with a constant brushing pattern under constant pressure for a specified period of time. Typodonts were then photographed and the images analyzed to quantify removal of plaque.

Plastic adult typodont models (Baistra Medical Instruments, Zhengzhou, China) were evenly coated with simulated plaque comprising either a green marker spray (Occlude^TM^, Pascal, Bellevue, WA, USA) and Glogerm^TM^ or a fluorescent marker. These indicators were found to be suitable for use as simulated plaque during validation studies. Coated typodonts (up to 8 per experiment) were mounted onto a brushing simulator (model ZM-3.8, SD Mechatronik, Feldkirchen-Westerham, Germany, Figure 1). Unused soft, full-head multi-level toothbrushes (Colgate-Palmolive Company) were mounted on the brushing simulator and used with a pressure of 200 g for 30 s. using a zig-zag motion at 106 strokes per minute. These parameters were determined to be the most suitable for consistent removal of simulated plaque during validation studies.

### Simulated Plaque Removal

Three molars on each of three typodonts were coated with simulated plaque, as described above. The occlusal surfaces of the molars were brushed, as described above. Relevant top-down images were captured and analyzed as above, before, and after treatment. Mean levels of simulated plaque remaining on surfaces with or without brushing were compared.

### Removal of Simulated Plaque; Water vs. Dentifrice

Three molars on each of three typodonts were coated with simulated plaque, as described above. Typodont sections were mounted on the brushing simulator such that the facial surfaces could be brushed across the teeth, distally to medially. Toothbrush heads were saturated in distilled water or a 1:3 slurry of a 1,450 ppm fluoride dentifrice in a silica base (FTP) before treatment and analysis as described above. Mean levels of simulated plaque remaining on surfaces treated with water or FTP were compared.

### Development of an *in vitro* Model to Cultivate Dental Plaque on Typodont Surfaces

**Figure 2:**
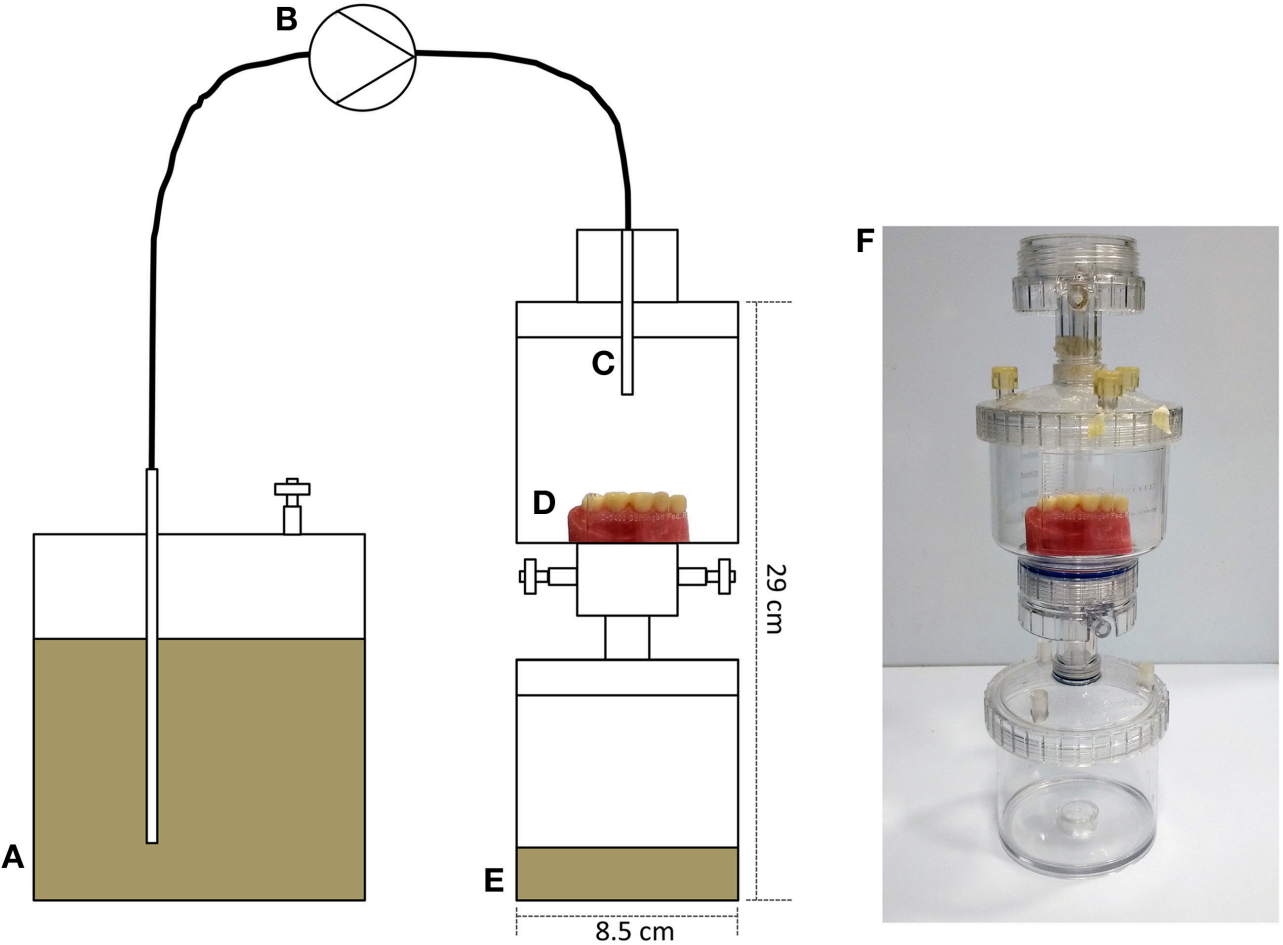
Development of a custom-built drip-flow model for the cultivation of dental plaque on typodont surfaces. **(A)** Medium vessel containing artificial saliva; **(B)** peristaltic pump running at approximately 4 mL h-1; **(C)** capillary delivering medium, drop-wise, onto a plastic typodont surface **(D)** in a sterile housing comprising a growth vessel and a waste vessel **(E)**. **(F)** Shows an image of the assembled model. The model was run for 72 h at 37°C and surfaces were inoculated daily with the saliva of a healthy adult volunteer (2 mL). Visible plaque was evident following this growth phase.

Polycarbonate vacuum-filter units (Sartorius, Goettingen, Germany) were adapted for use as sterile housings in which to contain typodont sections and a waste vessel (Figures 2A–F). A medium vessel containing artificial saliva medium (mucin, 2.5 g/L; bacteriological peptone, 2.0 g/L; tryptone, 2.0 g/L; yeast extract, 1.0 g/L; NaCl, 0.35 g/L; KCl, 0.2 g/L; CaCl2, 0.2 g/L; cysteine hydrochloride, 0.1 g/L; hemin, 0.001 g/L; and vitamin K1, 0.0002 g/L) was attached via a peristaltic pump running at ~4 mL h^−1^. Medium was delivered drop-wise onto the typodont for 72 h at 37°C and surfaces were inoculated daily with the saliva of a healthy adult volunteer (M, 34 years, 2 mL).

### Removal of Dental Plaque;—New vs. Used Brush

Dental plaques were cultivated on typodonts using the drip-flow model described above. Surfaces were immersed in a plaque disclosing solution (Tri Plaque ID gel, GC, Alsip, IL, USA) diluted 1:2 in distilled water, resulting in plaque stained in pink or purple. Stained typodont sections were mounted on the brushing simulator such that occlusal surfaces could be brushed, as above. Two types of brush were mounted on the simulator; new or artificially worn. All toothbrush heads were saturated in distilled water before use. Relevant top-down images were captured and analyzed, as above, before and after treatment and mean levels of plaque remaining on surfaces after brushing were quantified.

### Image Capture and Analysis

Simulated plaque on treated and untreated typodonts was visualized under controlled light conditions using a digital SLR camera (D3200 with an AF-S DX Micro NIKKOR 40 mm f/2.8G lens, Nikon, Tokyo, Japan). Removal of simulated plaque was indicated by absence or reduction of green coloration. Using image analysis software (ImageJ, National Institutes of Health, Bethesda, Maryland, USA); each of three top-down images were converted to grayscale and the mean light signal was calculated for the total occlusal surface or interproximal region of each of three molars (unless stated otherwise).

### Statistical Analyses

Differences between treatments were assessed using the student's *t-*test.

### Ethical Approval

Advice was taken from the Chair of a University of Manchester Research Ethics Committee regarding the correct procedures associated with the use of human saliva samples for the *ex vivo* experiment. The committee granted exemption from formal ethics approval due to the nature of the work, but as advised, written informed consent was obtained from all volunteers and all samples were collected anonymously.

## Results and Discussion

The current study describes the development and use of model system in which typodonts, coated with simulated plaque or microbial biofilms of oral origin were used to assess the effectiveness of cleaning following defined hygienic regimens controlled using a computerized brushing simulator. This was done using the artificial plaques Occlude^TM^ (an indicator powder spray with recommended applications that include intra oral dental articulation marking) and Glogerm^TM^ (a fluorescent powder) or by growing plaque biofilms derived from freshly collected saliva continuously fed with artificial saliva on typodonts held within an aseptic housing. An advantage of the use of *in vitro* models over human volunteer studies is that confounders such as poor volunteer compliance, variation between volunteers in brushing practices or duration can be eliminated.

**Figure 3:**
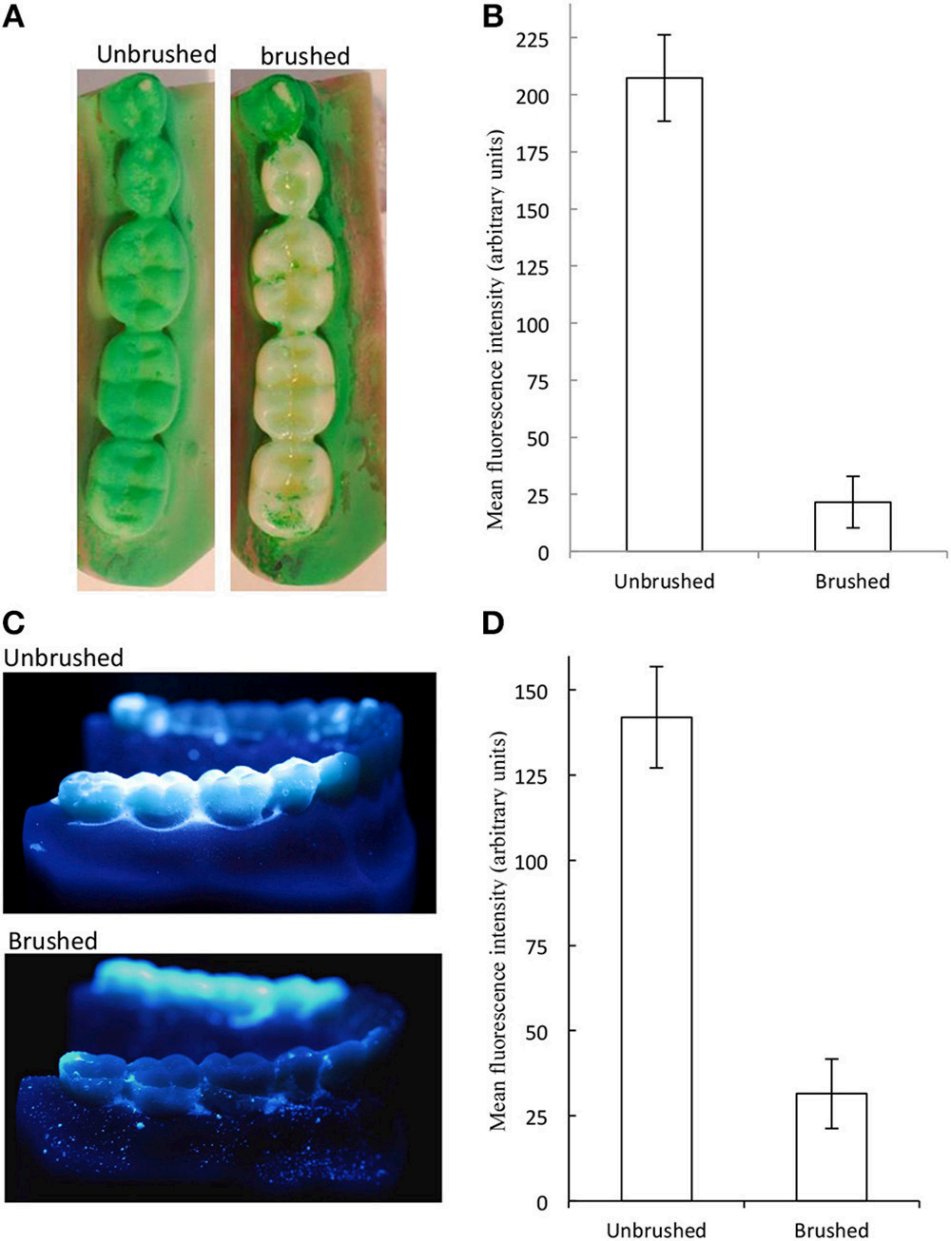
Brushing caused significant changes in simulated plaque. The molars of plastic typodonts were coated with simulated plaque and brushed with an unused toothbrush under constant pressure and brushing pattern. Simulated plaque was visualized under controlled light conditions. Plaque removal is indicated by absence/reduction of green coloration **(A)** and quantification (mean and standard deviations) **(B)** for occlude and by fluorescence **(C,D)**. Significantly less plaque was detected on brushed surfaces (*p* < 0.01, *n* = 9 typodonts).

Initially, the well-documented and substantial cleaning effect of brushing could be quantified and was demonstrated using image analyses of typodonts. Data presented in Figure 3 show a representative image (Figure 3A) and data (Figure 3B) derived from image analyses of replicated experiments using Occlude^TM^ indicator and Figures 3C,D show a representative image and data, respectively, for Glogerm^TM^ fluorescent indicator. These data indicate that brushing of the occlusal surfaces of the first, second and third molars with water and an unused soft, full-head multi-level toothbrush brush for 30 s with a pressure of 200 g, resulted in a highly significant (88%) removal of simulated plaque (*p* < 0.01). Whilst biofilms, including dental plaque are notoriously difficult to eradicate where they are inaccessible (37) effective cleaning through physical disruption by brushing is a universally accepted process in dental hygiene (38, 39). Thus, data in Figure 3 illustrate the general efficacy of physical cleaning in removing biofilms from surfaces where they are accessible such as the occlusal surfaces of molars. The images support this observation but also indicate that isolated pockets of simulated plaque persisted within pits and fissures suggesting that brushing for longer than 30 s. and utilizing dentifrice would result in improved plaque removal. Indeed, it is recommended by the American Dental Association that teeth should be brushed twice a day with gentle force for at least 2 min, using a fluoride-containing dentifrice (40), which may additionally include antimicrobial compounds (41, 42). Whilst typodonts were brushed for 30 s. in the current study in contrast to the recommended 2 min for optimal oral hygiene, this was focused on the occlusal surfaces only.

**Figure 4:**
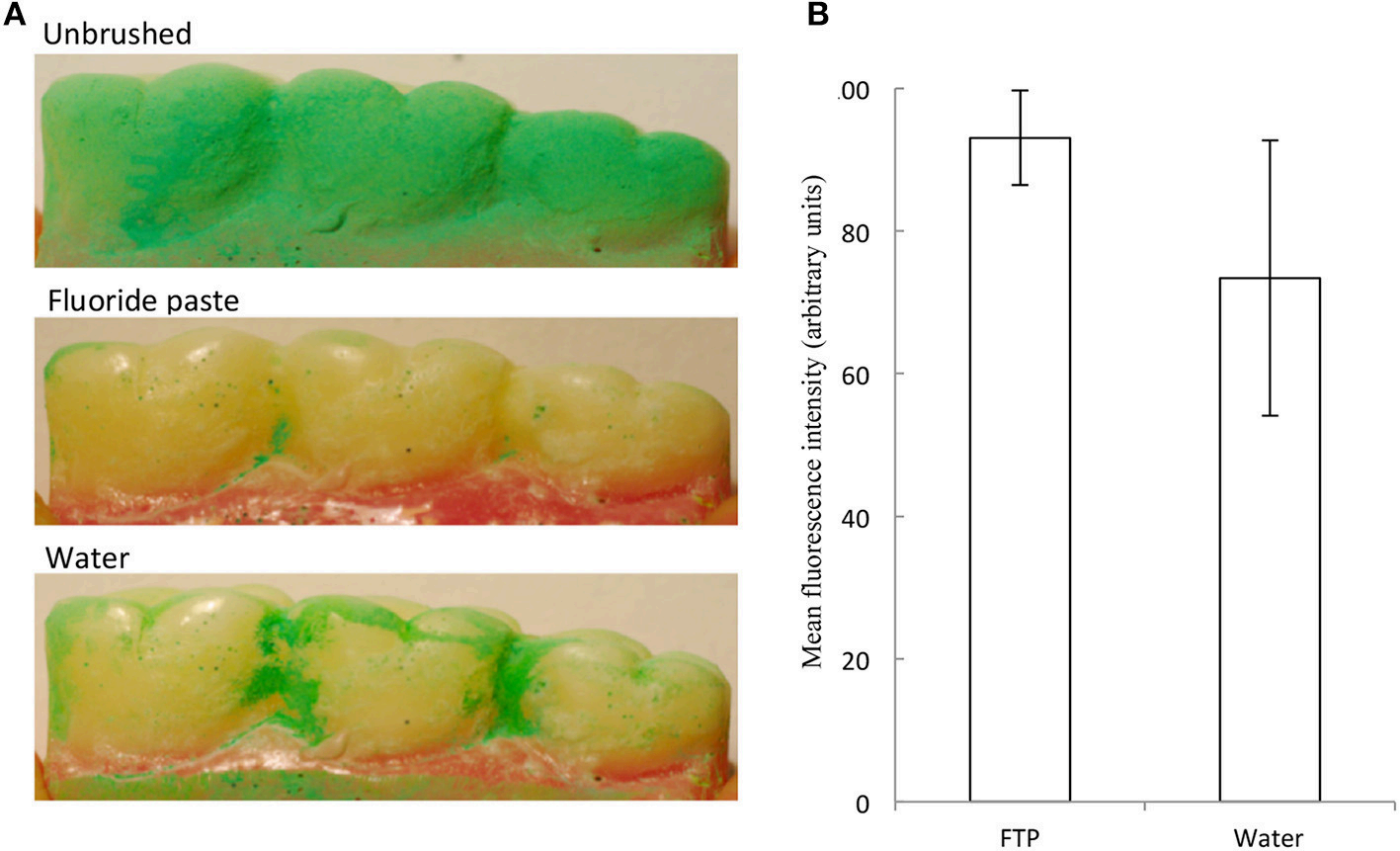
A fluoride toothpaste (FTP) removed more simulated interproximal plaque than water. Example images are shown. Representative images **(A)** and data (mean and standard deviations) **(B)** show removal of simulated plaque from the interproximal regions. FTP removed significantly more simulated plaque than water (12 separate experiments) (*p* = 0.017; *n* = 12).

Since there is evidence in the literature regarding the benefits of using dentifrice (40, 43) we utilized the controlled and reproducing brushing regimens facilitated by the brushing machine to assess this without the variability associated with manual brushing. An experiment was carried out by brushing with a dentifrice vs. brushing with water on the removal of Occlude^TM^ or Glogerm^TM^ on tooth cleaning. Since cleaning of the molar occlusal surfaces described above was highly effective in the absence of dentifrice, the interproximal areas of the buccal sides of the first, second, and third molars were selected as a more challenging surface to clean. Based on experiments that were repeated independently 12 times, the use of dentifrice achieved significantly greater cleaning (c 20%) than did brushing with water alone (Figures 4A,B). This finding agrees with a previous study (44). The additional cleaning effect is likely to be due to cleaning compounds present in the dentifrice (45), together with surfactant-specific effects such as intra-oral dispersion and micellization of hydrophobic ingredients.

The American Dental Association recommends that toothbrushes should be replaced every 3–4 months to maximize plaque removal, but the effect of brush wear on plaque removal has previously been difficult to assess. Warren et al. (34) conducted a clinical investigation showing that new toothbrushes were significantly more effective at removing plaque than those with significant wear and an additional report by Conforti et al. (35) supports this observation. In contrast however, Hogan et al. (46) reported that a worn powered toothbrush head did not impede the effectiveness of plaque removal.

**Figure 5:**
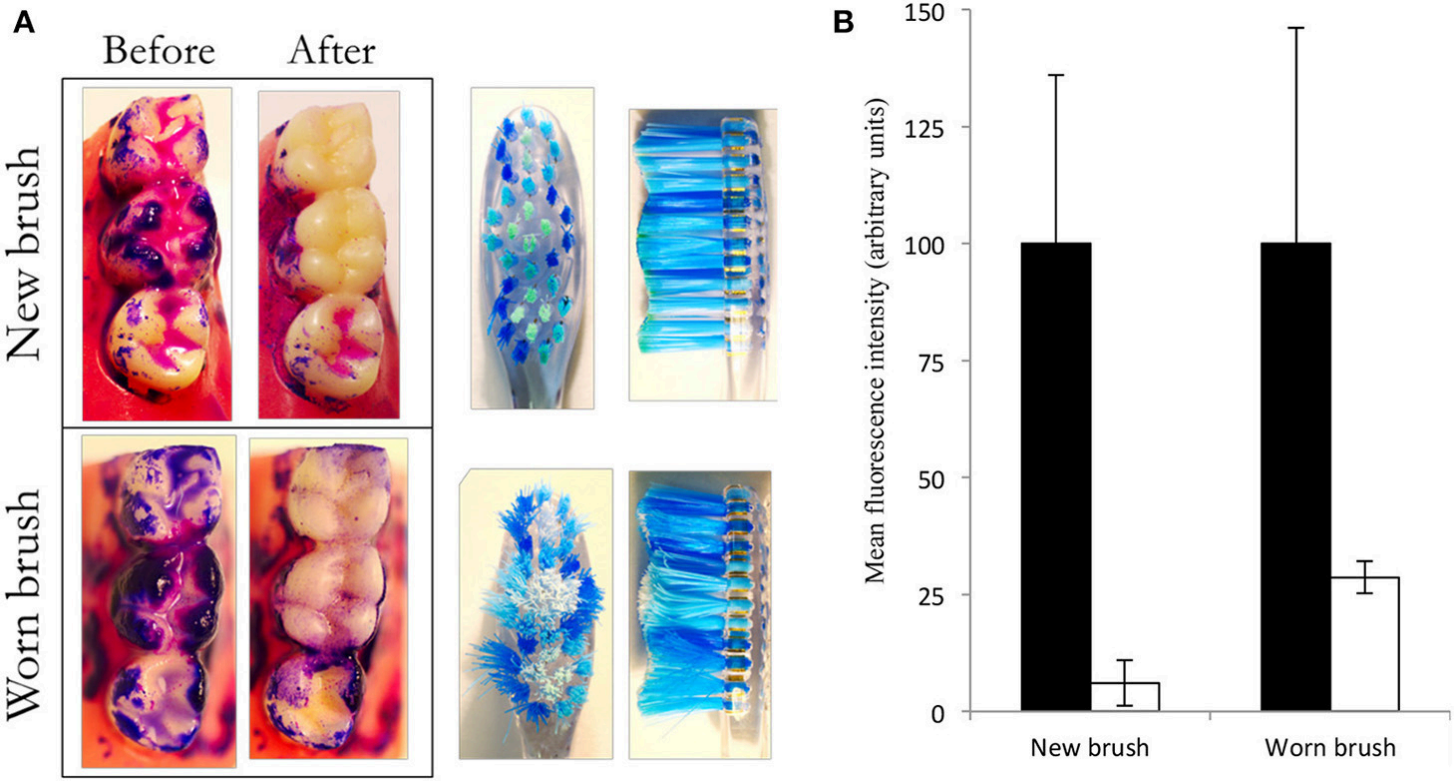
New brushes remove more plaque than worn brushes. Oral biofilm was cultivated, stained and brushed with a wetted toothbrush (new or worn) under constant pressure and brushing pattern. Images show stained plaque before and after brushing **(A)**. The red channel was isolated, converted to gray-scale and inverted to differentiate between areas with or without plaque. The occlusal surfaces of two molars were isolated and light intensity was measured using ImageJ. Data show background-corrected mean signal intensity. Higher numbers indicate higher levels of plaque. Black bars represent plaque levels before brushing, while light bars represent plaque levels after brushing. Error bars show standard deviations **(B)**. Data represent two separate experiments; mean values and standard deviations. Both brush types caused significant reductions (*p* < 0.05). The new brush removed significantly more biofilm than the worn brush (*p* < 0.05; *n* = 6 typodonts).

To develop the model to better represent the diversity and complexity of *in situ* dental plaque, a system was developed in which typodonts, supported in an aseptic housing can be inoculated with fresh saliva and fed continuously drop-wise with artificial saliva to grow salivary biofilms on the occlusal surfaces of the three molars as shown in Figure 5A. Previous data generated using similar feeding systems (47–49) suggest that considerable plaque accumulation can be achieved at solid-air interphases. Once developed, typodont plaques were stained with a plaque disclosing solution and brushed with water and brushes that had been mechanically worn to simulate 3 months of previous use. Image analyses showed that the new brushes achieved significantly (*p* < 0.05) greater plaque removal than the worn toothbrush than did the worn brushes (93.9 and 71.4%, respectively; Figure 5B). This is likely to be due to the fraying of the bristles with wear (35).

With respect to limitations of the current study, we focused on typodonts representing adult dentition, and applied the brushing regimens specifically to facial and occlusal surfaces of the molars.

Whilst caries in molar fissures is prevalent in adults, it is particularly so in children. Therefore, a similar study utilizing typodonts representing the dental anatomy of children would also be of relevance to dental hygiene. The current study established a methodological approach that could be used to address range to oral hygiene applications. A follow-on study could therefore consider the removal of plaque from the facial surfaces and the gingival margins of a range of teeth, including molars and incisors. Such analysis would have relevance for periodontal disease as well as caries.

## Conclusion

In summary, a model utilizing typodonts and simulated or biological plaque, together with standardized brushing parameters reproducing some characteristics of *in situ* dental plaque was used to evaluate the effect of various oral hygiene protocols. Data generated indicate that (i) plaque removal by standardized brushing regimens could be visualized and quantified; (ii) that plaque removal was significantly augmented by the addition of a toothpaste; and (iii) that brush head wear significantly reduced the effectiveness of brushes for the removal of biofilms developed from mixed oral bacteria. Whilst we have focussed on the occlusal surfaces of molars, the method could be applied other tooth surfaces and materials, and could incorporate the use of a viability indicator (50) to assess both plaque removal and bacterial inactivation. The model system, applying toothpastes at in-use concentrations may have advantages over *in vitro* methods that use lower concentrations since concentration exponents of antimicrobial compounds can vary markedly (51) and physical and antibacterial effects could be assessed concomitantly. The study illustrates the utility of the brushing simulator to investigate oral hygiene regimens.

## Author Contributions

RL: co-wrote the manuscript and experimental design. JL: co-wrote the manuscript and data generation. SF: supervision of laboratory work. JP: data generation. PS: assisted in experimental design. AM: overall supervision of experimental design, data generation, and manuscript preparation.

### Conflict of Interest Statement

PS is an employee of Colgate-Palmolive. The remaining authors declare that the research was conducted in the absence of any commercial or financial relationships that could be construed as a potential conflict of interest.
